# Impalement upon a Pitchfork-Handle, Entering per Vaginam

**Published:** 1854-01

**Authors:** 


*Impalement upon a Pitchfork-handle, entering per Vaginam. Recovery.*

[At the meeting of June 13th of the Boston Society for Medical Improvement, Dr. Sargent, of Worcester, related the following extraordinary case, which occurred in his practice, nearly two years ago.]

A lady, of about 37 years of age, who had borne several children, the last about three years previous to the injury about to be mentioned, and whose last menstrual period had been about a week before, her bowels also being in good lax condition, in sliding down from a hayloft, impaled herself upon the handle of a pitchfork, which passed in at her vagina *to the length of twenty-two inches*, when her feet struck the ground. The handle was immediately withdrawn, the patient carried into the house, and Dr. S. sent for. He found the patient, half an hour after the injury, lying on her back, with the thighs flexed, and the skin cool, pale, and moist (as if from fright,) and the pulse not much accelerated. There was no external injury, and no physical evidence of effusion into abdomen or thorax, and no urine nor fæces on the garments, nor about the person, nor on the field of the accident, nor on the handle of the fork. There was some blood flowing from vagina. Patient passed water during the visit, and it was not stained with blood. She complained most of pain in the left thorax, on a line with the scapula. Dr. S. saw the handle of the fork, which was rounded, a little larger at the end than otherwhere, perfectly smooth, two inches in diameter, and showed distinctly the stain of blood up to an abrupt line, twenty-two inches from the end.

Dr. S. theorized, in this case, that the instrument must have perforated the vagina at its upper part to the left, and gone between the uterus and rectum. [If it had gone to the right it would have perforated the cæcum.] The form of the instrument would make it much easier for it to pass be-between than to perforate organs, and Dr. S. *supposed that it passed in front of the kidney, behind the spleen and between the diaphragm and*

*false ribs, peeling up the costal pleura till it reached the scaleni muscles.* The subsequent history of the case, which *showed a fracture of the first rib*, while also, there was at no time any effusion into the chest, proved this diagnosis correct. Supposing that the greatest safety of the patient was in what might be called *forced rest*, Dr. S. gave her one grain of morphia (by estimate), and bound her chest firmly with a broad bandage of new flannel, placing a towel, wet in cold water, between this and the skin. The morphia was repeated in an hour, and one-third of a grain three hours after. Patient passed water repeatedly in first twenty-four hours, without trouble and without blood, and passed coagula from the vagina. The day following, there was emphysema above left clavicle; and, the day following, crepitus in left axilla high up, as if from fracture of bone. There was at no time any evidence of pneumonia or pleurisy, though there was deficiency of respiratory murmur in left chest from the pain in its expansion, the percussion remaning good.

The pulse stood at 120 for several days, and the opiates were continued aboutas long.

The injury was inflicted the 7th of August, 1851, and Dr. S. was in daily attendance for nine days; and, occasionally, afterwards, for three weeks. The recovery was entirely favorable, the patient being left only with an ill-united fracture of the first rib, over which there was some painful swelling for several weeks, which ultimately subsided, leaving an osseous prominence in the supra clavicular region, in intimate relations with the scaleni muscles.—*Am. Jour. of Med. Sciences*, October.

[In another part of the same Journal, we find the following somewhat similar case, communicated by Prof. Meigs, and reported by Dr. G. S. Bryant, formerly of Virginia.]

"During my residence in Amherst county, Va., in 1850, I was called, on the 25th of April, at about 3 P. M., to see Phœbe, a slave, at 25, black, smooth skin, small stature, and the mother of three healthy children.

"On arrival, learned that, at about 2 P. M., patient had leaped from the height of ten feet, and alighted upon a tobacco stick, which had been driven firmly in the ground and was concealed by some loose fodder. The stick was four and a half feet long, and one inch square. The vagina was entered without doing much injury to the vulva; the stick passed up the canal, and perforated its walls on the right side of the os uteri, entered the cavity of the abdomen, and passed in an oblique direction upward, and finally lodged against the twelfth and eleventh ribs of the right side.

"4 P. M. Hæmorrhage quite subsided, but at the time of accident it was very profuse from vagina; pulse 120, and very small; extremities cold; countenance anxious; pain in abdomen distressing; nausea and frequent vomiting; mind clear.

"*Treatment.*—R. Tinct. opii ℥j; brandy ℥ij. To be given at once, and repeated every hour or two until reaction, or relief was obtained; warm applications to the extremities, and a poultice to the entire abdomen, constituted the principal treatment.

"26*th*, 4 P. M. Slept during the latter part of last night, and has been sleeping, occasionally, during the morning, but is not altogether free from pain. Reaction took place about 12 o'clock last night; pulse now 110, quick and hard; abdomen much swollen, hard, and tender to touch; complains a good deal of the side, about the point where the stick lodged, and the lower region of the liver. The swelling and contusion externally are considerable, and she cannot bear the part to be handled; vulva very much inflamed; passes water with much pain and difficulty.

"Dover's powder, grs. x, at bedtime, to be repeated during the night if necessary; effervescing draught every two hours; continue poultices.

"27*th*, 10 A. M. Rested pretty well last night; pulse 112, hard; skin dry; abdomen very much distended and painful to touch; eyes very red; has vomited some bilious matter; passes her water still with difficulty; bowels have not been moved since accident. R. Hyd. chlo. mit. grs. vj; rhei, grs. x. Make iv pills; to be given at once, and followed by an enema of soap and water in six or eight hours, if no action is had by this time; anodynes and poultices continued; vulva to be frequently cleansed with Castile soap and warm water.

"28*th*, 11 A. M. Pulse 100, and softer; has had several bilious discharges; some discharge of pus from vagina; no other material change. R. Blue mass, grs. xvj; Dover's powder, grs. xi. Make into viij pills. One to be given every six hours. Continue effervescing draught, poultices, &c.

"29*th*, 10 A. M. Abdomen enormously distented, dull on percussion and painful on pressure; bowels have been moved twice, discharges of bilious character; pulse 118, small and quick; rested badly last night; skin dry, tongue coated over with a brown fur. Continue treatment.

"30*th*, 10 A. M. Had, about 2 o'clock last night, a copious discharge of grumous blood from the bowels, which discharge continued to occur every hour or two until 9 A. M., this morning; could not ascertain the exact quantity; nurse supposed it to be from seven to eight quarts; *this is no doubt a too liberal estimate.* Abdomen has gone down very much; pulse 130, small and feeble; skin dry and cool; she seems quite exhausted; vaginal discharge continues. Ordered half a grain of sulph. morphia at once; infusion of serpentaria ℥j, to be given at intervals of two hours. Continue pills and poultices, but discontinue draught.

"*May* 2*d*, 9 A. M. Abdomen much flattened; had two bilious discharges yesterday, free of blood; pulse 112, small and soft; vaginal discharge more profuse; passes her water freely; skin dry; has some appetite, Continue treatment.

"4*th*, 10 A. M. Has done well since last visit, until last night. Nurse thinks she was alarmed by a conversasion which took place in the room upon the subject of death and her probable recovery. After an hour or two she was better, and again expressed her belief that she would get well, never before having any doubt about her recovery. Bowels have been moved once this morning; biliary secretions improving; skin continues dry; pulse 108; appetite better. Continue treatment; is allowed a more nutritious diet.

"6*th*, 10 A. M. Pulse 108, soft; skin moist; bowels in good condition; appetite good; vaginal discharge diminishing; complains of little else than soreness in the right side.

"Ordered tonics and better diet; mercury discontinued; no appearance whatever of its constitutional effects.

"8*th*, 12 M. Convalescing. Continue tonics.

"11*th*, 11 A. M. Convalescing rapidly.

"Recovered fully by the middle of June following."

